# Stabilization of Antioxidant and Anti-Inflammatory Activities of Nano-Selenium Using *Anoectochilus burmannicus* Extract as a Potential Novel Functional Ingredient

**DOI:** 10.3390/nu15041018

**Published:** 2023-02-17

**Authors:** Pensiri Buacheen, Angkana Chaipuang, Jirarat Karinchai, Onanong Nuchuchua, Arisa Imsumran, Ariyaphong Wongnoppavich, Nuttaporn Pimpha, Pornsiri Pitchakarn

**Affiliations:** 1 PhD Program in Biochemistry, Faculty of Medicine, Chiang Mai University, Chiang Mai 50200, Thailand; 2 Department of Biochemistry, Faculty of Medicine, Chiang Mai University, Chiang Mai 50200, Thailand; 3 National Nanotechnology Center, National Science and Technology Development Agency, Thailand Science Park, Pathum Thani 12120, Thailand

**Keywords:** nanomedicine, selenium, orchid, cryoprotectant, lyoprotectant, stabilizer

## Abstract

*Anoectochilus burmannicus* is an orchid that contains phenolic compounds and exhibits antioxidant and anti-inflammation properties. This study aimed to investigate whether its ethanolic extract (ABE) can be used as a reducing agent and/or a stabilizer of nano-selenium (SeNP) synthesis. SeNPs exhibited higher antioxidant activity than ABE-SeNPs. In contrast, ABE-SeNP (4 µM Se) had greater anti-inflammatory activity in LPS-induced macrophages than SeNPs. Interestingly, ABE acted as a stabilizer for SeNPs by preventing particle aggregation and preserving its antioxidant activity after long-term storage (90 days). Moreover, after the freeze-drying process, ABE-SeNPs could be completely reconstituted to suspension with significantly stable antioxidant and anti-inflammatory activities compared to freshly prepared particles, suggesting the cryoprotectant and/or lyoprotectant role of ABE. The present study shows the potential of ABE as an effective stabilizer for nanoparticles and provides evidence for the development of ABE-SeNPs as a food supplement or novel functional ingredient for health benefits.

## 1. Introduction

Selenium (Se) is an essential trace element with various vital roles in human health. It is an important component of selenoenzymes, including glutathione peroxidase, thioredoxin reductases, and iodothyronine deiodinases. These enzymes are required in many physiological processes of the human body, such as the antioxidant defense system, thyroid hormone formation, immune system, cancer protection, and reproduction [1]. Insufficient intake of Se can impair the antioxidant system and lead to the risk of aging and various illnesses such as cardiovascular disease and thyroid disease [2,3]. Moreover, inadequate intake of Se can cause Keshan disease and Kashin–Beck disease, which are well-known to be related to Se deficiency [4,5]. In the past decades, Se has been used as a supplement for not only Se-deficient individuals but also for the prevention or treatment of metabolic-related disorders such as hyperlipidemia, atherosclerosis, hypercholesterolemia, and hyperglycemia [6]. Selenomethionine (SeMet), an organic form, is the most common species of Se in the dietary supplement because the absorption and retention of the organic form are higher than with the inorganic forms, including selenate (SeO_4_^2−^) and selenite (SeO_3_^2−^) [7,8]. There is, however, a concentration limit between the beneficial effects and the toxicity with respect to living organisms. Selenium nanoparticles (SeNPs) have attracted interest for use as food supplements or therapeutic agents because of their low toxicity with better bioavailability compared with traditional selenium supplementation. A previous study has shown that the administration of various selenium forms in rats led to different degrees of intercellular disruptions in the liver tissue. The largest disruptions are caused by selenate, followed by selenite, whereas small damages were found in selenium nanoparticle (SeNP)-treated rats, suggesting that nanoparticle synthesis can be used to reduce the toxicity of selenium [9].

A nanoparticle, a material in the nanoscale, is widely used in biomedical industries for drug and gene delivery as well as a food supplement. There are various advantages, such as high carrier capacity, high mobility, cellular uptake, improved bioavailability, lower toxicity, and several routes of administration [10]. SeNPs can be synthesized by various physical, chemical, and biological methods. The simple process of SeNP synthesis occurs through a chemical reduction reaction using ascorbic acid as a reducing agent to form nanometer-sized particles with the zero-oxidation state. In addition, SeNP synthesis by chemical reaction usually forms in the presence of polysaccharides, which be used as effective stabilizers [11]. One study has shown that stabilizing the nanoparticle with chitosan prevents its aggregation, and the stabilized nanoparticle exhibits higher antidiabetic activity than that of SeNPs [12]. Moreover, surface decoration by mushroom polysaccharides–protein complexes present a highly efficient way to enhance the cellular uptake and anticancer efficacy of nanomaterials [13]. These pieces of evidence suggest that using surface capping agents on SeNPs could improve their stability and biological activities. Recently, plant extracts have been widely employed for the green synthesis of nanoparticles as an eco-friendly method. The extracts that contain phenolic or flavonoid compounds such as garlic, parsley leaves, green tea, and ginger extracts are usually used as reductants in SeNP synthesis [14,15,16,17]. Furthermore, natural compounds can be also used as stabilizers and not only as reducing agents. For example, surface-modified SeNPs with curcumin showed higher stability and cellular uptake than non-modified SeNPs [18]. 

Many biological activities of *Anoectochilus* species, including *A. formosanus* and *A. roxburghii*, have been previously reported, such as antioxidant, antihyperglycemic, antihyperliposis, and hepatoprotective activities [19,20,21,22]. These orchid species have been widely used in traditional medicine [23]. They have been reported to contain kinsenoside, a major active component [24]. This study focused on the use of *A. burmannicus* extract (ABE), which contains high phenolic content, and can be found in Thailand and other countries such as Bhutan, Laos, China, Vietnam, and Indonesia. A study by Budluang et al. found that an aqueous extract of *A. burmanicus* can exhibit antioxidant and anti-inflammatory as well as anti-insulin resistance activities [25]. Moreover, the ethanolic extract also showed antioxidant and inflammatory activities [26]. Due to the antioxidant property and high phenolic content of ABE, this study hypothesized that ABE can be used as a reducing agent and/or a stabilizer for selenium nanoparticle synthesis, and it may enhance both physical and biological properties of the nanoparticle. This study provides substantial evidence that may be beneficial for new alternative selenium supplementation, which is safer and provides higher efficacy than current forms of Se-NP.

## 2. Materials and Methods

### 2.1. Preparation of Anoctochillus burmannicus Ethanolic Extract (ABE)

*A. burmannicus* orchids were cultivated by tissue culturing at the Queen Sirikit Botanic Garden, Chiang Mai, Thailand. The whole plant samples were washed and dried in a hot-air oven at 60 °C for 24 h and kept at −20 °C. The extraction procedure was previously described by Karinchai et al. [26]. Briefly, a dried whole plant of *A. burmannicus* was powdered and soaked in 80% ethanol at the ratio of 10:100 *w*/*v* with occasional shaking and stirring. The ethanolic fraction was filtered and evaporated using a rotary evaporator at a temperature of 50 °C. Next, the extract was freeze-dried by lyophilization to obtain crude *A. burmannicus* ethanolic extract powder (ABE). The ABE powder was kept at −20 °C until use.

### 2.2. Synthesis of Anoectochillus burmannicus Ethanolic Extract-Synthesized Selenium (ABE-SeNPs)

ABE-SeNPs were synthesized according to Menon et al. and Liu et al. with slight modification [17,27]. In brief, a sodium selenite (Na_2_SeO_3_) solution was dissolved in water to prepare a 0.1 M Na_2_SeO_3_ solution. Then, the Na_2_SeO_3_ solution (0.3 mL) was mixed with 10 mg/mL of ABE (28.5 mL) and stirred at 600 rpm for ten minutes. To investigate whether ABE itself can be a reductant for the synthesis, the mixture was mixed without ascorbic acid for 120 h (five days). For another condition, ascorbic acid was suggested to be used as an initiator of the reduction process; the solution of 0.1 M ascorbic acid (1.2 mL) was added dropwise into the mixture and stirred for six hours. The unreacted materials including the extract, ascorbic acid, and Na_2_SeO_3_ were removed by dialysis against Milli-Q water at 4 °C for 24 h. Moreover, SeNPs were also synthesized by the same method, but the solution of ABE was replaced with an equal volume of Milli-Q water. A brick red color solution represents the success of SeNP synthesis.

### 2.3. Characterization of ABE-SeNPs

The physical properties of SeNPs and ABE-SeNPs were characterized. The morphology of the nanoparticles was observed using transmission electron microscopy (TEM) at an accelerating voltage of 120 kV (Hitashi; HT7800 Series 120 kV). Size and zeta potential were determined by dynamic light scattering analysis (DLS, Ultro Pro, Malvern) with a Zetasizer Nano instrument. Each sample was dried and then ground into a homogeneous powder to record the infrared spectra on a Thermo Scientific Nicolet iS50 FT-IR spectrometer with a built-in diamond attenuated total reflection (ATR). The spectra were acquired at 400–4000 cm^−1^ wavenumbers with a 4 cm^−1^ resolution with sample scanning 32 times. The concentration of the nanoparticles was determined using inductively coupled plasma analysis (ICP-OES hydride generation, Perkin Elmer, 7300 DV, USA).

### 2.4. Antioxidant Activity by ABTS Assay

The ABTS^+^ scavenging ability was determined as described in Budluang et al. [25]. Briefly, 7 mM ABTS^+^ stock solution was mixed with potassium 2.45 mM (1:1, *v*/*v*) persulfate and left in the dark at room temperature for 12–16 h until the reaction was complete and the absorbance was stable. The ABTS^+^ reagent was diluted with DI H_2_O to an absorbance of 0.700 ± 0.02 at 734 nm (Microplate reader Synergy Hybrid Reader, BioTek). The nanoparticle solution (100 µL) was mixed with the ABTS^+^ solution (100 µL) in multiple 96-well plates. Deionized H_2_O was used as a control. The absorbance was read at 734 nm after the reaction every 15 min until 60 min. The antioxidant activity was calculated as % inhibition using the following equation.

$${\mathrm{ABTS}}^{+}\mathrm{scavenging}\mathrm{effect}(\%)=\frac{\left(\mathrm{Absorbance}(\mathrm{control})-\mathrm{Absorbance}(\mathrm{sample})\right)}{\mathrm{Absorbance}(\mathrm{control})}\times 100$$

### 2.5. Cell Line and Cell Culture

A RAW 264.7 murine macrophage-like cell line was obtained from American Type Culture Collection (ATCC). The cells were cultured as a suspension culture in an ultra-low attachment culture dish in DMEM with L-glutamine supplemented with 10% FBS and 1% penicillin under 5% CO_2_ at 37 °C. The cells were harvested and subjected to experiments when the cells reached 80% confluence.

### 2.6. Cytotoxicity Assay

RAW 264.7 macrophages were plated at 1.5 × 10^4^ cells/well (96-well). The cells were treated with various doses of SeNPs, ABE-SeNPs, or Na_2_SeO_3_ (10–25 µM of selenium concentration) for 48 h. To determine cytotoxicity, a sulforhodamine B (SRB) assay was performed. The treated cells were fixed with 10% (*w/v*) trichloroacetic acid and stained with SRB for 30 min, after which the excess dye was removed by washing repeatedly with 1% (*v*/*v*) acetic acid. The protein-bound dye was dissolved in 10 mM Tris-base solution for determination of absorbance at 510 nm using a microplate reader. The percentage of cell survival was calculated following the equation below. A non-toxic or sub-toxic concentration of the nanoparticles was then used for further experiments.

$$\mathrm{Cell}\mathrm{viability}(\%)=\frac{\mathrm{Absorbance}(\mathrm{sample})}{\mathrm{Absorbance}(\mathrm{control})}\times 100$$

### 2.7. Anti-Inflammatory Activity by Measuring Nitric Oxide Production

RAW 264.7 cells were seeded (2.5 × 10^4^ cells/well) in a 96-well plate for 12 h. Next, the cells were pre-treated with SeNPs, ABE-SeNPs, or Na_2_SeO_3_ (1.0, 2.0, 4.0 µM of selenium concentration) for one hour, and then inflammation was further induced by LPS for 24 h. Then, the culture medium was collected to determine nitric oxide (NO) production using the Griess reagent. The nitrite concentration was determined by measuring the absorbance of the azo color at 540 nm by a microplate reader. The amount of nitric oxide was determined in comparison with the sodium nitrite standard curve. The experiment was independently performed three times with the value represented as mean ± SD.

### 2.8. Immunoblot Analysis

Treated-RAW 264.7 cells were collected and lysed with RIPA buffer containing protease inhibitors. The protein samples were subjected to 10% SDS-PAGE and transferred to a nitrocellulose membrane. The target proteins were probed with specific antibodies, an anti-iNOS antibody at a dilution of 1:2000 (Merck, Rahway, NJ, USA), and an anti- β-actin antibody at a dilution of 1:10,000 (Sigma, St. Louis, MO, USA). Protein detection was carried out using a chemiluminescence reagent.

### 2.9. Stability of ABE-SeNPs

The stability of ABE-SeNPs was determined by solubility observation and measurement of diameters and zeta potential as well as antioxidant ability analysis after storage of the nanoparticles at 0, 15, 30, 60, and 90 days at 4 °C compared to those of SeNPs.

### 2.10. Reconstitution of ABE-SeNPs

Storage of nanoparticles in their solid form can extend their shelf-life. Poor solubility is a limitation when the solid nanoparticle is reconstituted into a suspension. To investigate whether ABE could improve nanoparticle reconstitution, the samples including SeNPs and ABE-SeNPs were freeze-dried by lyophilization to obtain the nanoparticle powder. Then, the particles were reconstituted with DI-H_2_O and subjected to the observation of solubility and the measurement of their physical properties, including size and zeta potential, and their biological properties, namely, antioxidant and anti-inflammatory activities.

### 2.11. Statistical Analysis

All values were given as mean ± standard derivation (SD) from triplicate samples of three independent experiments. Overall, the differences among the treatment groups were determined using a one-way analysis of variance (ANOVA) followed by Tukey’s multiple comparison test using Prism 9.0 software. *P* values < 0.05 were regarded as a measure of statistical significance.

## 3. Results 

### 3.1. Synthesis and Characterization of Selenium Nanoparticles 

**Figure 1:**
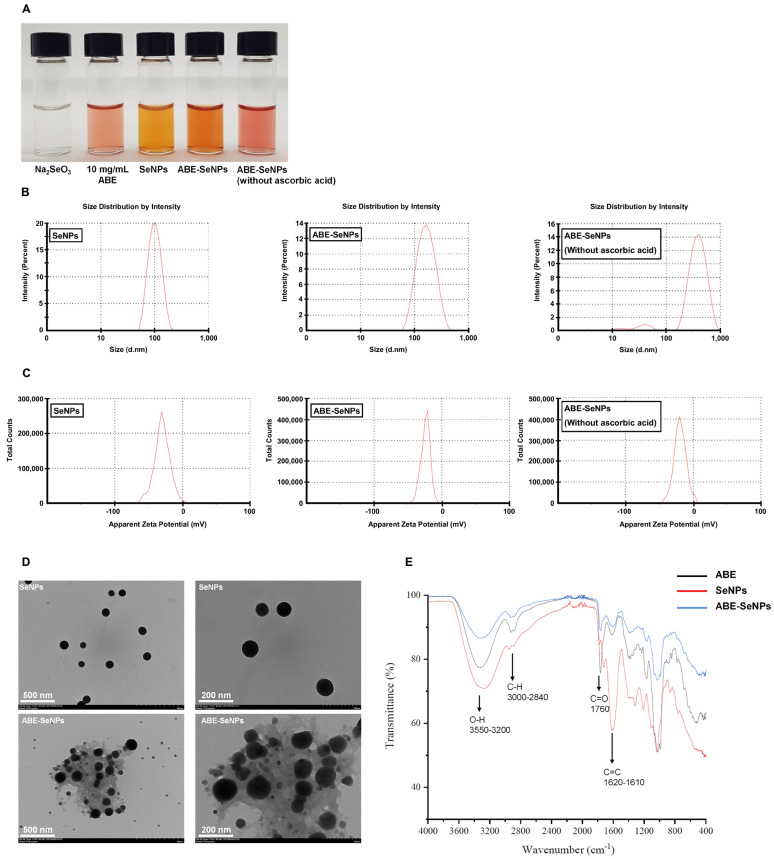
Physical properties of SeNPs and ABE-SeNPs. Color of the selenium salt, ABE, and the nanoparticle solution (**A**), size distribution (**B**), zeta potential (**C**), and morphology of SeNPs and ABE-SeNPs observed by TEM (**D**), and FTIR spectra of ABE, SeNPs, and ABE-SeNPs (**E**).

Both SeNPs and ABE-SeNPs were synthesized by a general chemical method using ascorbic acid as a control. To investigate whether ABE could be a reducing agent for nanoparticle synthesis, ABE-SeNPs were synthesized in the absence of ascorbic acid (Figure 1A). Physical properties were measured including size (Figure 1B), PDI, and zeta potential (Figure 1C) of the nanoparticles. The average diameter of SeNPs was 95.1 ± 6.43 nm, whereas that of ABE-SeNPs obtained from the synthesis with ascorbic acid was 145.0 ± 3.39 nm. Yet, ABE-SeNPs formulated without ascorbic acid showed the largest size, namely, 331.73 ± 10.11 nm (Figure 1B). PDI and zeta values of SeNPs and ABE-SeNPs formulated with and without ascorbic acid were 0.087 ± 0.046, 0.366 ± 0.074, and 0.589 ± 0.203, respectively, and −30.9 ± 0.4, −24.5 ± 1.9, and −21.2 ± 1.6 mV, respectively (Figure 1C). For use in nanomedicine, a particle size of less than 200 nm is suggested to avoid lymphatic system activation [28]. Moreover, optimized sizes of 50–200 nm in diameter provide a great cellular uptake rate [29]. In the case of pharmaceutical nanoparticles, a PDI of 0.3 and below is desired [30]. Therefore, the results suggest that the optimal condition for the synthesis of ABE-SeNPs should employ ascorbic acid as an initiator for the step of the reduction process. ABE-SeNPs synthesized with ascorbic acid were subjected to further study. The Se concentrations of SeNPs and ABE-SeNPs analyzed by ICP analysis were 665.95 ± 39.59 and 620.57 ± 5.48 µM, respectively.

The morphology of the nanoparticles observed by transmission electron microscopy (TEM) analysis was found to be spherical (Figure 1D). Interestingly, ABE-SeNPs showed the background surrounding the nanoparticles, but that was not observed in SeNPs, suggesting that selenium nanoparticles might be enclosed/wrapped with organic compounds of ABE. In addition, Fourier transform infrared spectroscopic analysis (FTIR) confirmed the capping of ABE on the surface of selenium nanoparticles, as shown in Figure 1E. The FTIR spectrum of ABE showed multiple peaks at 3332 (OH group), 2925 (aliphatic C-H), 1766 (carbonyl C=O stretch), 1614 (C=C stretch), 1169 (C-O ester), and 11,031 (S=O sulfoxide) cm^−1^. In the FTIR spectra of SeNPs, the strong peak in the range of 3550–3200 cm^−1^ corresponded to the hydroxy groups of ascorbic acid-stabilized SeNPs. The O-H stretching showed a shift from 3265 to 3318 cm^−1^ in the presence of ABE [31,32]. There was a medium peak that was found in both spectra of ABE and ABE-SeNPs at 2925 and 2922 cm^−1^, respectively, but was not present in SeNPs. When comparing ABE-SeNPs and SeNPs, the high intensity of C=O and C=C bands suggests that more lactone structure of ascorbic acid exists in SeNPs than ABE-SeNPs. Moreover, the spectrum of ABE and ABE-SeNPs at the frequencies ranging between 1200 and 1400 cm^−1^ showed the same pattern, whereas some peaks were not detected in SeNPs, suggesting that ABE was incorporated with selenium nanoparticles.

### 3.2. Determination of the Antioxidant Activity of ABE, Sodium Selenite, and Selenium Nanoparticles by ABTS Assay 

**Figure 2:**
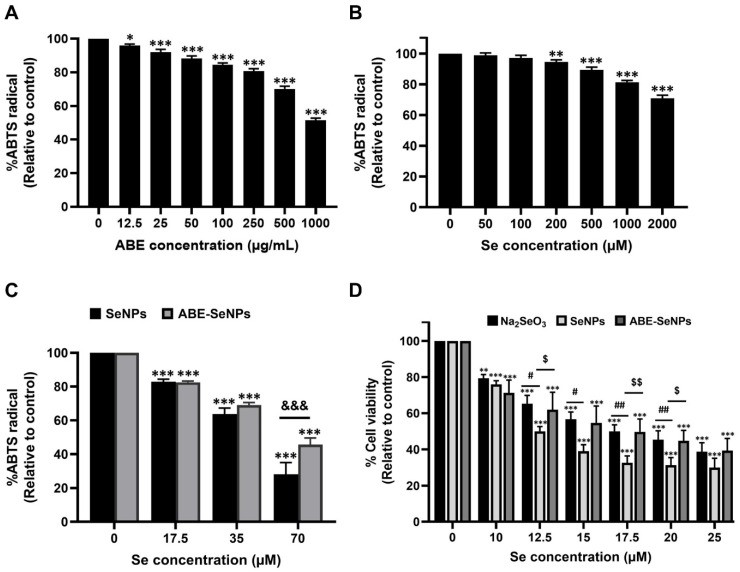
Antioxidant activity and cytotoxicity of ABE, selenium and Se nanoforms. ABTS+ scavenging activity of ABE (**A**), selenium (**B**), and Se nanoforms (SeNPs and ABE-SeNPs) (**C**). Cell viability of RAW 264.7 macrophages treated with Na_2_SeO_3_, SeNPs, and ABE-SeNPs for 48 hours (**D**). (The data are indicated as mean ± SD of three independent experiments: * *p* < 0.05, ** *p* < 0.01, *** *p* < 0.001 compared to control (non-treated group), &&& *p* < 0.001 SeNPs compared to ABE-SeNPs, # *p* < 0.05, ## *p* < 0.01 compared to Na_2_SeO_3_, $ *p* < 0.05, $$ *p* < 0.01 SeNPs compared to ABE-SeNPs.).

The antioxidant activity of sodium selenite, SeNPs, ABE-SeNPs, and ABE was then assessed by the ABTS assay, which is based on the relative ability of antioxidants to scavenge the ABTS cation radical (ABTS^+^). ABE at the highest dose (1000 µg/mL) inhibited the ABTS^+^ radical at approximately 49% (Figure 2A). The selenium salt exhibited poor antioxidant activity (Figure 2B) when compared to the nanoform (Figure 2C). SeNPs and ABE-SeNPs (70 µM of Se) significantly inhibited the ABTS^+^ radical levels by approximately 70% and 55%, respectively. The antioxidant activity was markedly improved by the reduction of sodium selenite to the nanoparticle form. ABE did not show any additive or synergistic effect on SeNPs, which might be due to the ABE concentration incorporated into the particle being under its effective dose.

### 3.3. Cytotoxicity of Sodium Selenite and Selenium Nanoparticles (SeNPs and ABE-SeNPs) on RAW 264.7 Macrophages

In this study, the cytotoxicity of sodium selenite and selenium nanoparticles (SeNPs and ABE-SeNPs) was performed by an SRB assay, which is based on the ability of a dye to bind protein basic amino acid residues in the living cells. RAW 264.7 macrophages were treated with various concentrations of selenium for 48 h. Figure 2D shows that the non-toxic concentration of Se was less than 10 µM. Sodium selenite and ABE-SeNPs decreased the cell viability by 50% (inhibition concentration 50; IC50) at 17.5 µM of Se concentration, while the IC50 of Se in the SeNP form was 12.5 µM. SeNPs were more toxic than the salt form, which might be due to an increase in the cellular uptake [33], whereas the presence of ABE (ABE-SeNPs) could significantly reduce the cytotoxicity of SeNPs. The non-toxic concentration of Se was then further used to investigate the anti-inflammatory activity. 

### 3.4. The Anti-Inflammatory Effect of Selenium Nanoparticles Assessed by Nitric Oxide Production Assay in LPS-Induced RAW 264.7 Macrophage

**Figure 3:**
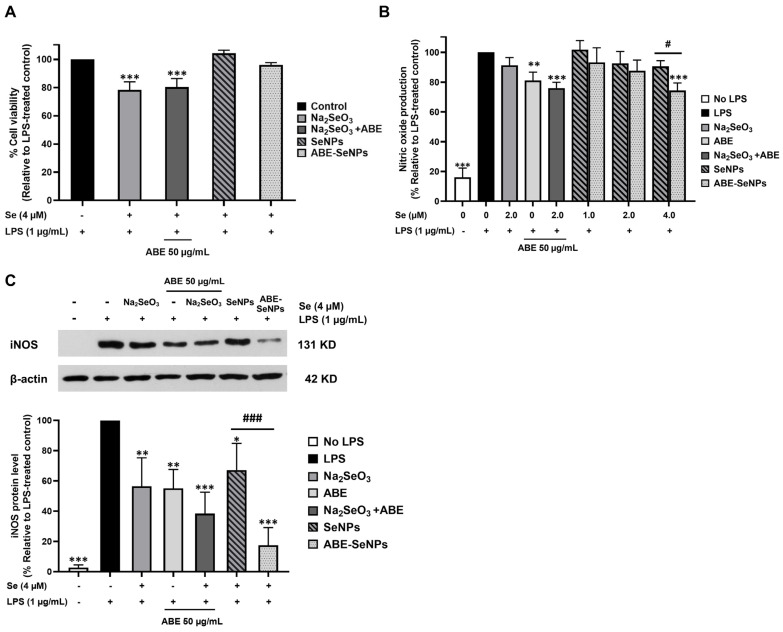
ABE-SeNPs exert anti-inflammation activity. Cell viability of RAW 264.7 macrophages treated with Na_2_SeO_3_, SeNPs, and ABE-SeNPs under the LPS-induced inflammatory condition for 24 h (**A**). Effect of Na_2_SeO_3_, ABE, Na_2_SeO_3_ +ABE co-treatment, SeNPs, and ABE-SeNPs on nitric oxide production (**B**) and iNOS protein level (normalized with β-actin) (**C**) in LPS-treated RAW 264.7 macrophages (* *p* < 0.05, ** *p* < 0.01, *** *p* < 0.001 compared to LPS-treated group, # *p* < 0.05, ### *p* < 0.01 SeNPs compared to ABE-SeNPs, the data indicated as mean ± SD of three independent experiments).

NO overproduction is considered an abnormal condition in the cells, especially macrophages. Thus, it can be used as a marker to investigate the anti-inflammatory ability of a test compound. Most antioxidant agents can exhibit anti-inflammatory activity. Then, the anti-inflammatory effect of the nanoparticle was investigated in RAW264.7 cells. Lipopolysaccharide (LPS) was used to induce inflammation in the cells. Interestingly, it was found that when co-treated with LPS, Se at 4 µM showed higher toxicity to the cells than that of the nanoform (SeNPs and ABE-SeNPs) (Figure 3A).

As shown in Figure 3B, NO production was slightly but not significantly inhibited by the single treatment of sodium selenite as well as SeNPs at an equal concentration (2 µM). ABE-SeNPs at 2 µM and 4 µM Se significantly decreased NO levels by 13% and 25%, respectively. The activity of ABE-SeNPs was higher than that of SeNPs, suggesting that the incorporation of ABE into SeNPs could enhance the anti-inflammation ability of SeNPs. Interestingly, the combination treatment between sodium selenite (2 µM) and ABE (50 µg/mL) significantly decreased NO production by 23%, suggesting the additive effect of ABE on Se. The combination treatment of Se and ABE was more efficient than ABE-SeNPs because of possible limited ABE content that interacted with selenium nanoparticles in ABE-SeNPs. Furthermore, the protein level of iNOS, a key enzyme generating nitric oxide, was investigated by Western blotting. Consistent with the result of nitric oxide production, the reduction of the iNOS protein level by ABE-SeNPs was significantly greater than that of SeNPs (Figure 3C). These results suggest that the synthesis of SeNPs with ABE could improve the anti-inflammation activity of the nanoparticle. Moreover, Se in the nanoforms (both SeNPs and ABE-SeNPs) can prevent LPS-induced cytotoxicity in the macrophages.

### 3.5. Stability of SeNPs and ABE-SeNPs 

**Figure 4:**
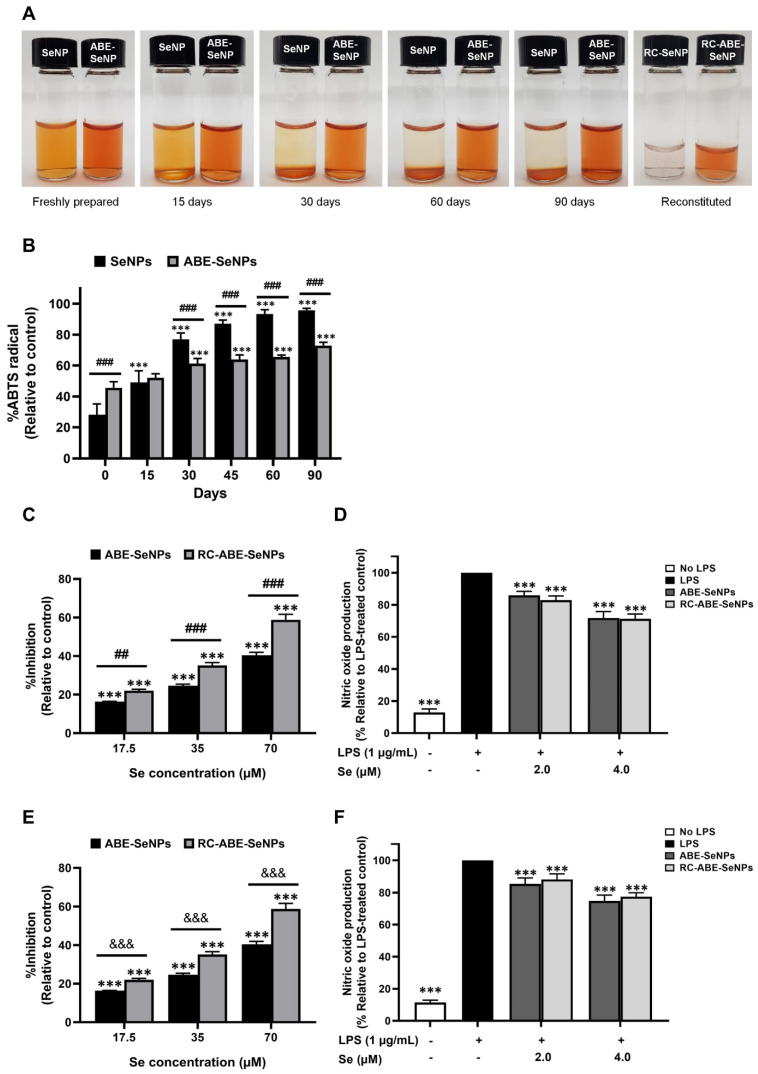
Stability of ABE-SeNPs is greater than SeNPs. Visual observation of SeNPs and ABE-SeNPs after fresh preparation, different times of storage, and reconstitution (**A**). Free radical scavenging activity of SeNPs and ABE-SeNPs (Se concentration, 70 µM) determined by ABTS assay after storage for 15, 30, 60, and 90 days at 4 °C (**B**). ABTS radical scavenging activity (**C**), and anti-inflammatory ability investigated by nitric oxide production (**D**) of ABE-SeNPs and RC-ABE-SeNPs. Free radical scavenging (**E**), and anti-inflammation (**F**) activities of ABE-SeNPs and RC-ABE-SeNPs after storage at 4 °C for 30 days. (*** *p* < 0.001 compared to control, ## *p* < 0.01, ### *p* < 0.01 SeNPs compared to ABE-SeNPs, &&& *p* < 0.001 ABE-SeNPs compared to RC-ABE-SeNPs. The data indicated as mean ± SD of three independent experiments).

Stabilization of SeNPs is a strategy that has been of major concern for its application. The nanoparticles tend to aggregate into large clusters, resulting in lower bioactivity, bioavailability, and biocompatibility. Surface-capping agents on SeNPs could improve their stability and biological activity [34,35]. This experiment determined whether ABE could enhance SeNP stability. SeNPs and ABE-SeNPs were stored at 4 °C for various times, and then their physical properties and antioxidant abilities were investigated. After storage at the indicated times, the physical properties of SeNPs and ABE-SeNPs were slightly changed by increasing the size and decreasing the zeta value (Table 1). The aggregated particles of SeNPs settled to the bottom after storage for 15 days, and the solution was almost colorless after 60 days of storage (Figure 4A). The changes in SeNPs were remarkable after storing SeNPs for 90 days. Interestingly, the precipitate was hardly observed in stored ABE-SeNPs until 90 days, despite physical changes (Figure 4A). It can be suggested that ABE could stabilize the nanoparticle by agglomerate-particle prevention. Although freshly prepared SeNPs (70 µM Se) provided higher antioxidant ability than those of ABE-SeNPs, the antioxidant activity of ABE-SeNPs was significantly greater than SeNPs when the particles were in long-term storage (30–90 days) (Figure 4B). ABE-SeNPs retained the antioxidant capacity (48% inhibition), while the activity of SeNPs was significantly decreased 1.4-fold (from 70% to 50% inhibition) when stored for 15 days. The activity of SeNPs continuously decreased in long-term storage (30–90 days), whereas ABE-SeNPs showed greater activity than that of SeNPs, although their activity was decreased by two-fold (From 54% to 27%) after 90 days of storage. These results suggest that ABE has the potential to be a stabilizer for selenium nanoparticle synthesis. 

### 3.6. Cryo/Lyoprotectant Properties of ABE

To extend the shelf-life of nanoparticles, storage in the solid form has been suggested [36]. Compared to the suspension form, the solid form can be stabilized by avoiding physical changes. A nanoparticle solution or suspension can be converted into solids by a freeze-drying process known as lyophilization. However, freezing and drying steps could generate various stresses, leading to physical changes. A capping agent or stabilizer was employed to protect the nanoparticle from stress and improve the solubility when reconstituted. This study, therefore, examined whether ABE acts as a cryo/lyoprotectant of SeNPs. Freshly prepared SeNPs and ABE-SeNPs were subjected to lyophilization to obtain a powder or solid form. Next, the powder of the nanoparticles was reconstituted with DI-water, and then the suspensions were measured for physical properties as well as biological activity. ABE-SeNPs were completely aqueous soluble, whereas SeNPs showed aggregated particles and were non-homogeneous (Figure 4A). These results were consistent with the physical properties of the nanoparticles analyzed by DLS, as shown in Table 2. Reconstituted-SeNPs (RC-SeNPs) dramatically showed physical changes by increased size and lower zeta value compared to the freshly prepared SeNPs, whereas the zeta value of reconstituted-ABE-SeNPs (RC-ABE-SeNPs) was not significantly changed compared to the freshly prepared form, although the particle size was increased. Next, the biological activities of RC-ABE-SeNPs were further determined, as shown in Figure 4C,D. Both the antioxidant and anti-inflammatory activities of RC-ABE-SeNPs remained and were not significantly different from the freshly prepared ABE-SeNP. Moreover, these activities of RC-ABE-SeNPs were preserved after 30-day storage in a solid form (Figure 4E,F). These results suggest that ABE can play a role as a cryo/lyoprotectant by preventing stress during the freezing and drying processes of SeNPs.

## 4. Discussion

In this study, we established ABE-SeNPs synthesized by an ascorbic-redox system. It exhibited antioxidant and anti-inflammatory activities. The physical and biological properties of ABE-SeNPs showed more stability than those of SeNPs, suggesting ABE as an efficient stabilizer. Moreover, ABE-SeNPs could be reconstituted into suspension form without changing both physical properties and biological activity.

Plant extracts can be applied as reducing agents and/or stabilizers for green synthesis because they contain various bioactive compounds, including phenolic compounds, polysaccharide, and other metabolites, which are rich in antioxidants [37,38]. In the present study, we focused on *A. burmannicus* ethanolic extract because it contains phenolic compounds and exerts biological activities, including antioxidants [26], and it may be useful as a reducing agent and/or a stabilizer for nano-selenium synthesis. The achievement of selenium nanoparticle synthesis was observed by a color change due to the surface plasmon resonance effect. The control condition showed that the colorless solution was changed to orange color after adding ascorbic acid, suggesting the selenium nanoparticle was synthesized (Figure 1A). In the presence of ABE, the success of nanoparticle synthesis could not be visually observed because the color of the extract interfered with the red brick color of the SeNP solution. The maximum absorption wavelength assessed by UV-spectrophotometry showed the lambda max of ABE was at 262 nm, similar to SeNPs (data not shown/supplement data). According to the size and PDI data, we found that the suitable condition for ABE-SeNPs synthesis required ascorbic acid to initiate the redox reaction of selenium, suggesting that ABE is not a strong reducing agent, or the ABE concentration used in the synthesis is insufficient. The average size of SeNPs synthesized by the chemical method has been reported in a wide range from 5 to 200 nm. Various factors impact particle size, including the molar ratio of elemental selenium, the reducing agent and stabilizer, the time of reaction, as well as temperature [12,39,40]. A previous study reported that the average size (analyzed by SEM and DLS) of SeNPs synthesized by fenugreek seed extract was slightly larger than that of the chemical method [41]. Like our study, the nanoparticle size of ABE-SeNPs (~145 nm) was larger than SeNPs (~95 nm). The increasing diameter of ABE-SeNPs could indicate that ABE is incorporated with the nano-selenium. Moreover, the image obtained from TEM analysis showed a thin film background of ABE-SeNPs, indicating ABE wrapped around the surface of the particles. Likewise, the nano-selenium using *Vitis vinifera* extract showed a thin film encapsulating the nanoballs, confirming the presence of a polymeric layer covering the nanoballs [42]. Normally, zeta potential is used to predict the stability of nanoparticles. An absolute value equal to or greater than ± 30 mV is considered stable [43]. In the present study, the zeta value of the nanoparticles showed negative charges. The value was −30.9 ± 0.4 and −24.5 ± 1.9 mV in SeNPs and ABE-SeNPs, respectively, suggesting that SeNPs are considered more stable than ABE-SeNPs. Conversely, the stability testing demonstrated that ABE-SeNPs were more stable than SeNPs. ABE may help to delay particle aggregation and improve antioxidant activity. The decreasing zeta value of ABE-SeNPs might be caused by the capping of ABE on the nanoparticle surface. Together with the results that showed a larger size and lower zeta value, ABE-SeNPs might be stabilized by the steric hindrance effect of ABE. It is the phenomenon of the slowing of chemical reactions due to steric bulk [44]. Moreover, storage conditions such as temperature, lighting, and time could affect nanoparticle stability by changing physical properties, resulting in agglomerate particles and loss of function [45,46]. 

Lyophilization is a water removal process typically used to preserve perishable materials, extend shelf life, or make the material more convenient for storage and transport. Thus, the long-term stability of nanoparticles could be extended by storing them in solid form. However, the stress generated during the freezing and drying step impairs the physical properties of the nanoparticle, resulting in destabilization and insolubility [36]. Capping agents on the nanoparticle surface including a polymer (polyethylene glycol) or sugar (sucrose, glucose, trehalose, mannitol) can protect against stress from the freeze-drying process [47,48,49]. These capping agents act as a cryoprotectant and lyoprotectant to protect against damage from the freezing and the dehydration steps, respectively. In addition, using cryoprotectants along with a stabilizer such as a chitosan prolongs storage duration and improves its reconstitution in water [49]. Our study found that lyophilized ABE-SeNPs were completely resuspended in DI water, but SeNPs were not. Moreover, the antioxidant and anti-inflammatory activities of ABE-SeNPs after reconstitution were not different from the freshly prepared particles. After storage for 30 days, physical properties as well as anti-inflammation activity did not decline when ABE-SeNPs were stored in solid form. Interestingly, RC-ABE-SeNPs stored for 30 days exhibited greater antioxidant ability than that of the suspension of 30-day-storage ABE-SeNPs. These data suggest that dry storage of the nanoparticles extends their shelf-life, and ABE exhibits the potential to be a stabilizer as well as a cryoprotectant and/or lyoprotectant for SeNPs. 

The bioactive compounds as well as organic components in the crude ABE extract might play a key role in stabilizing the nanoparticle. The profile of *Anoectochilus* species exhibits the contents of polysaccharides and other bioactive compounds, including lactone glycosides (kinsenoside and its diastereoisomer, goodyeroside A), flavonoids, and organic acid [50,51]. A previous study reported that polysaccharide improves the stabilization of Se nanoparticles synthesized in a redox system using ascorbic acid. The presence of chitosan stabilized the nanoparticle for up to 60 days without the aggregated particle compared to bare SeNPs [12]. Moreover, there were numerous studies using polysaccharides from plant extracts to stabilize nano-selenium, such as *Dictyophora indusiate* [52], *Rosa roxburghii* fruit [53], *Spirulina platensis* [54], *Polyporus umbellatus* [55], and *Sargassum fusiforme* [32]. In addition, Xiguang et al. found that green tea nanoaggregates could be used as a templet to stabilize SeNPs due to polyphenols in green tea infusions spontaneously assembling into nanoaggregates via the interaction with proteins, carbohydrates, and other components [56,57]. This evidence supports the possibility that polysaccharides together with other compounds contained in the crude extract of ABE stabilize the nanoparticle. 

Plant extracts or bioactive compounds not only stabilize the nanoparticles but also improve their biological activities. For example, curcumin-loaded SeNPs enhanced apoptosis and cell cycle arrest in cancer cells and tumor growth inhibition in mice models when compared to curcumin or bare SeNPs treatments [35]. It has been reported that the SeNP preparations using EGCG, a key bioactive compound in green tea, could improve the bioactivity of nano-selenium, but purified EGCG would markedly increase the cost of SeNPs-EGCG synthesis [56,58]. Then, green tea extract was used instead of the pure compound, and the nanoparticle synthesized with the extract exhibited a stronger inhibition effect on the proliferation of carcinoma cells than that of the Se salt form. Moreover, crude polysaccharides of *Sargassum fusiforme* stabilized SeNPs and significantly enhanced the antioxidant activity of the particles [32] A study done by Pandiyan et al. found that SeNPs synthesized using *Thymus vulgaris,* a medicinal plant, exhibited antioxidant and anti-inflammatory properties [59]. Moreover, crude extracts or whole plant extracts have been reported to have superior activity over the isolated single constituents through additive and/or synergistic effects of bioactive compounds [60]. This supports the idea to use crude extract for enhancing the biological activity of the nanoparticle. Our study found that both selenium nano forms, namely, SeNPs and ABE-SeNPs, showed higher anti-inflammation activity than the Se element by decreasing the nitric oxide level through the reduction of iNOS protein in inflamed cells. Using ABE in the synthesis of SeNPs did not show the additive effect as found in the combination treatment of ABE and Se element. This might be due to the limited amount of ABE capping on the nanoparticle surface. The ABE content was probably lower than its effective dose suggesting that the initial ABE concentration used for SeNP synthesis should be increased or the ABE-SeNP synthesis method must be further optimized to obtain a higher amount of ABE loading or capping on the particle surface. Interestingly, a low amount of ABE can stabilize SeNPs and preserve their biological functions. Thus, ABE can be used as a stabilizer for selenium nanoparticle synthesis to prevent physical property changes and to maintain biological activities.

Supplementation of selenium provides several benefits for health, including antioxidant system, cancer, cardiovascular disease, diabetes, and inflammation-associated diseases [61]. However, long-term or excessive intake as well as some chemical forms of selenium may cause toxicity to the human body [62,63]. Based on selenium forms, the toxicity of selenium could be reduced by the reduction of Se into the nanoparticle form. A previous study in a mice model reported that nanoforms of selenium are less toxic than inorganic and organic forms, as shown by less bone marrow cell death, less harm to liver tissue, and prevention of DNA damage in the animals treated with SeNPs [9,64]. Zhang et al. reported that the toxicity of nanoparticles stabilized by polysaccharides from *Spirulina platensis* was less than 30-fold compared to sodium selenite in RAW 264.7 macrophages [54]. In addition, previous studies showed that the toxicity of nanoparticle-based green synthesis as well as surface decoration with the natural compound is lower than sodium selenite in normal cell lines [65,66]. Moreover, antioxidant ability of the selenium nanoform has been reported to be higher than the elemental form [67]. Our study showed that the cytotoxic effect of SeNPs was higher than that of sodium selenite while surface modification with ABE could reduce the toxicity of the nanoparticles in RAW 264.7 macrophages. However, both forms of nano-selenium were not toxic to the cell under the inflammatory condition (LPS-treated cells) when compared to Se element single treatment or a combination treatment of Se and ABE, suggesting that nanoparticle formulation could reduce the toxicity of selenium and an LPS combination treatment. This probably is involved with the reduction of oxidative stress and/or inflammation in the cells by either SeNPs or ABE.

In conclusion, this study indicates that ABE has the potential to be a stabilizer for nano-selenium. It prevents physical property change and improves the biological activities of the SeNP suspensions. Moreover, it acts as a cryoprotectant and/or lyoprotectant during the freeze-drying process of the SeNPs, resulting in the complete resuspension of the particles with the preservation of both physical and biological properties. This application might be an alternative method of eco-friendly SeNP synthesis. However, the limitation of ABE capping-efficacy on the nanoparticle surface should be solved by the optimization of the synthesis method in future studies to obtain ABE-SeNPs that provide great efficiency of antioxidant and anti-inflammation abilities. A future study is important to determine the efficiency of the particles by measuring the cellular uptake and the effect of the nanoparticles on the cellular reactive oxygen species (ROS) level. Moreover, the bioavailability, pharmacokinetics, and safety in animals and the human body should be further investigated to develop ABE-SeNPs as a supplement or alternative pharmaceutical for nanomedicine. 

## Figures and Tables

**Table 1:** Size, PDI, and zeta potential of SeNPs and ABE-SeNPs after various 4 °C storage times.

Days	SeNPs			ABE-SeNPs		
	Size (nm)	PDI	Zeta (mV)	Size (nm)	PDI	Zeta (mV)
0	95.1 ± 6.43	0.087 ± 0.04	−30.9 ± 0.42	145.0 ± 3.39	0.366 ± 0.07	−24.45 ± 1.88
15	94.1 ± 1.99	0.192 ± 0.01 **	−31.8 ± 1.90	154.6 ± 9.82	0.404 ± 0.01	−20.48 ± 0.69 **
30	93.8 ± 4.72	0.166 ± 0.04 *	−28.8 ± 1.47	153.1 ± 21.47	0.369 ± 0.06	−20.50 ± 0.61 *
45	95.9 ± 5.33	0.186 ± 0.01 **	−25.0 ± 1.11 **	148.2 ± 12.72	0.369 ± 0.05	−19.40 ± 1.77 ***
60	102.8 ± 7.82	0.197 ± 0.02 ***	−24.8 ± 2.51 **	151.6 ± 15.04	0.392 ± 0.06	−20.58 ± 0.56 **
90	113.0 ± 10.50 *	0.229 ± 0.01 ***	−24.4 ± 2.90 **	148.5 ± 18.82	0.355 ± 0.07	−20.57 ± 0.75 **

The data indicated as mean ± SD of three independent samples; * *p* < 0.05, ** *p* < 0.01, *** *p* < 0.001 compared to day 0.

**Table 2:** Size, PDI, and zeta potential of SeNPs and ABE-SeNPs comparing freshly prepared and reconstituted forms.

Parameters	SeNPs		ABE-SeNPs	
	Fresh	Reconstituted	Fresh	Reconstituted
Size	97.1 ± 4.24	740.6 ± 138 ***	141.2 ± 8.90	244.0 ± 10.41 ***
PDI	0.069 ± 0.03	0.485 ± 0.13 ***	0.353 ± 0.07	0.422 ± 0.03
Zeta	−31.26 ± 0.37	−26.62 ± 0.71 ***	−24.01 ± 2.51	−23.17 ± 1.62

The data indicated as mean ± SD of three independent samples; *** *p* < 0.001 compared to freshly prepared.

## Data Availability

Not applicable.
